# Influence of Various Liquids on Characteristics of Backward A_1_ Lamb Wave in YX LiNbO_3_ Plate: Theory and Experiment

**DOI:** 10.3390/s26113516

**Published:** 2026-06-02

**Authors:** Andrey Smirnov, Ilya Nedospasov, Iren Kuznetsova

**Affiliations:** Kotelnikov Institute of Radio Engineering and Electronics of RAS, Moscow 125009, Russia; andre-smirnov-v@yandex.ru (A.S.); ianedospasov@mail.ru (I.N.)

**Keywords:** backward Lamb waves, interdigital transducer, acoustic impedance, permittivity, YX lithium niobate plate, finite element method, acousto-electronic liquid sensor

## Abstract

In this work, the effect of liquids with different dielectric permittivities and acoustic impedances on the characteristics of the backward antisymmetric A1 Lamb wave propagating in a YX LiNbO3 plate was investigated theoretically, numerically and experimentally for the first time. It was found that the dielectric constant and acoustic impedance (density) of a liquid make independent and separable contributions to measured parameters of interdigital transducers, such as the resonant frequency and Q-factor. It was shown that the backward A1 Lamb wave in a YX LiNbO3 plate can be effectively used as a basis for multiparametric liquid sensors. The results obtained are both of fundamental importance for understanding the physics of propagation of backward acoustic waves in piezoelectric plates with a liquid load and of applied value for the development of a new generation of acousto-electronic sensors based on such waves.

## 1. Introduction

Antisymmetric (An) and symmetric (Sn) Lamb acoustic waves and shear-horizontal (SHn) waves in piezoelectric plates have been actively investigated as a basis for various liquid sensors [1,2,3,4,5,6]. Lamb waves are characterized by different polarizations and, depending on the number of half-waves that fit along the plate thickness, can be of zero or higher orders (n = 0, 1, 2, …) [7]. It has been previously shown that Lamb acoustic waves in plates characterized by high radiation losses into a liquid can be used as liquid presence indicators [8]. Meanwhile, waves with a high electromechanical coupling coefficient can be used to create liquid conductivity sensors [9,10] or petrol identifiers [11]. There are also known works that have demonstrated the possibility of using acoustic waves with minimal radiation of acoustic energy into the liquid [12]. Thus, the feasibility of using direct acoustic waves in plates for creating liquid sensors has been convincingly demonstrated.

In recent years, backward acoustic waves in piezoelectric plates and structures have attracted considerable attention from researchers. These waves are characterized by oppositely directed phase and group velocities, as well as anomalous dispersion [13]. One of their unique features is the presence of a zero-group velocity (ZGV) point, whose frequency is strongly dependent on the surrounding medium [14,15,16]. In this regard, there currently exist a large number of publications proposing various sensors and nondestructive testing methods using the ZGV point [17,18,19,20,21]. It should be noted, however, that significantly less attention has been paid to the backward waves themselves, which exist at frequencies above the ZGV point. Nevertheless, these waves also exhibit high sensitivity to changes in the boundary conditions on the plate surface and can be used as a basis for various types of sensors [22,23]. Furthermore, the frequency range of their existence is larger than that of the ZGV point, which makes it possible to substantially simplify the fabrication technology of such devices.

It has recently been theoretically shown and experimentally confirmed that the backward antisymmetric Lamb wave in a YX lithium niobate plate does not transform into another wave and does not disappear in the presence of distilled water [24]. This opens up prospects for using backward acoustic waves for creating sensors of liquid properties.

It should be noted that the presence of a non-conducting and inviscid liquid on the surface of a piezoelectric plate leads to the appearance of acoustic loading and a change in the electrical boundary conditions due to the permittivity of the liquid. Furthermore, the change in the permittivity of the liquid loading leads to a redistribution of the electric field of the piezoactive acoustic wave in the region of the interdigital transducer. An analysis of the influence of these contributions on the properties of backward piezoactive waves in piezoelectric plates has not been previously conducted.

In light of the above, the present work presents a theoretical, numerical, and experimental study of the influence of liquids with various acoustic impedances and permittivities on the characteristics of the backward A_1_ Lamb wave in a YX lithium niobate plate in a wide frequency range.

## 2. Materials and Methods

### 2.1. Theoretical Study

#### 2.1.1. Transfer Matrix Method

**Figure 1:**
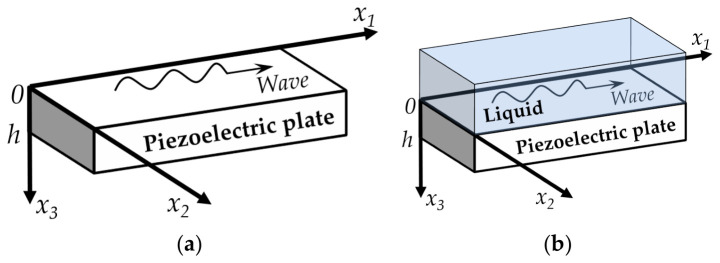
Geometry of the problem: (**a**) a free plate; (**b**) a plate loaded with liquid.

To calculate the phase velocity of the A_1_ Lamb wave in a piezoelectric plate in contact with an inviscid, non-conducting liquid, a boundary value problem was solved using an iterative algorithm together with the classical transfer matrix method [24]. The geometry of the structure under consideration is shown in Figure 1. The wave propagates in the plate along the *x*_1_ axis, bounded by the planes *x*_3_ = 0 and *x*_3_ = *h*. Two cases were considered for comparison. In the first case, the plate was in contact with a vacuum on both sides, i.e., for *x*_3_ < 0 and *x*_3_ > *h*. In the second case, the plate was loaded with a liquid in the region *x*_3_ < 0, while on the side *x*_3_ > *h*, it remained in contact with a vacuum. The problem was assumed to be two-dimensional, so all mechanical and electrical variables were considered to be constant along the *x*_2_ axis.

To solve the stated problem, the equation of motion, Laplace’s equation, and the constitutive equations for the piezoelectric medium were written down [25]:

(1) $$\rho \partial^{2}U_{i}/\partial t^{2}=\partial T_{ij}/\partial x_{j},\;\;\;\partial D_{j}/\partial x_{j}=0,$$

(2) $$T_{ij}=C_{ijkl}\partial U_{l}/\partial x_{k}+e_{kij}\partial \Phi /\partial x_{k},\;D_{j}={-\varepsilon }_{jk}\partial \Phi /\partial x_{k}+e_{ijk}\partial U_{l}/\partial x_{k}.$$

Here, *U_i_*, *T_ij_*, and *D_j_* are the components of the mechanical displacement of the particles, the mechanical stress, and the electrical displacement, respectively; *t*, *ρ*, *x_j_*, and *Φ* are the time, density, coordinate, and electrical potential, respectively; and $C_{ijkl}$, $e_{ikl}$, and $\varepsilon_{jk}$ are the elastic constants, piezoelectric constants, and permittivity, respectively.

In the region *x*_3_ > *h*, the electrical displacement must satisfy Laplace’s equation in both cases:

(3) $$\partial D_{j}^{V}/\partial x_{j}=0,$$

Here, $D_{j}^{V}=-\varepsilon_{0}\partial \Phi^{V}/\partial x_{j}$, index *V* denotes the values referring to a vacuum, and ε_0_ is the vacuum permittivity.

In the case of contact with a vacuum on the upper or lower free surfaces of the plate, standard boundary conditions corresponding to zero normal mechanical stresses were used.

In the case of the presence of a liquid in the region *x*_3_ < 0, harmonic waves in the inviscid liquid were described by the Helmholtz equation:

(4) $$\Delta u+k^{2}u=0,$$

where Δ, *u*, and *k* are the Laplace operator, particle velocity potential, and wave number, respectively.

At the liquid/plate interface (*x*_3_ = 0), the continuity conditions for the normal components of displacement and mechanical stress were used, as well as the absence of shear stresses on the solid surface from the liquid side. As electrical boundary conditions, the continuity conditions for the normal components of electric displacement and the electric potential were used.

The solution to the boundary value problem described above was sought in the form of plane inhomogeneous waves and was reduced to an eigenvalue problem [26,27].

#### 2.1.2. FEM Method

**Figure 2:**
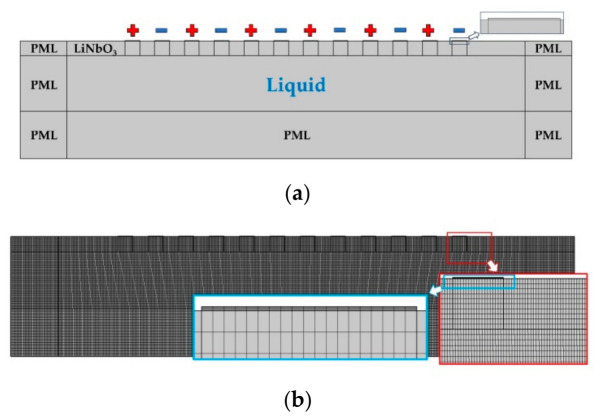
(**a**) Topology of the FEM model and (**b**) mesh used in the calculations.

Simulation of the excitation of acoustic waves in piezoelectric plates was performed using the finite element method in the COMSOL Multiphysics 5.3a environment. The geometry of the models corresponded to the experimental samples. Calculations were carried out in the frequency domain, and the magnitude of the *S*_11_ parameter was evaluated during the analysis. Modeling was performed for seven interdigital transducers (IDTs). Excitation of acoustic waves occurred via applying an alternating electric voltage to the electrodes. The wavelength (*λ*), equal to the IDT period, was varied from 0.9 to 2.7 mm in steps of 0.3 mm. Each IDT contained 6 pairs of aluminum strips, and the thickness of each strip was 500 nm. The set of IDTs was placed on the surface of a 350 μm thick Y-cut lithium niobate wafer. The width of each electrode and the distance between adjacent electrodes were *λ*/4. The topology of the FEM model and the mesh used in the calculations are shown in Figure 2. When constructing the computational mesh, rectangular elements were used, where the linear element size was chosen in the range from *λ*/5 (maximum) to *λ*/10 (minimum).

Mechanical loading from the electrodes was taken into account through the continuity of mechanical displacements and mechanical stresses at the metal/piezoelectric plate interface.

At the piezoelectric plate/vacuum interface, mechanical and electrical boundary conditions corresponding to a free surface were used. To suppress reflections from the edges of the plate, perfectly matched layers (PMLs) were placed along its perimeter.

In the case of the presence of a liquid on the plate surface, the mechanical boundary conditions used were the continuity of the normal components of displacement and mechanical stress, as well as the absence of shear stresses on the plate surface, as described in Section 2.1.1. The electrical boundary conditions in this case ensured continuity of the electric potential and the normal component of the electric displacement vector.

The thickness of the liquid layer was 10 times the plate thickness in the simulated structure. Additionally, to eliminate reflections from the boundaries of the computational domain, a PML was applied on the liquid surface, effectively absorbing waves traveling into the volume of the liquid.

The material constants of lithium niobate, given in Table 1 [24], were used in the calculation.

### 2.2. Experimental Study

#### 2.2.1. Experimental Sample Fabrication

**Figure 3:**
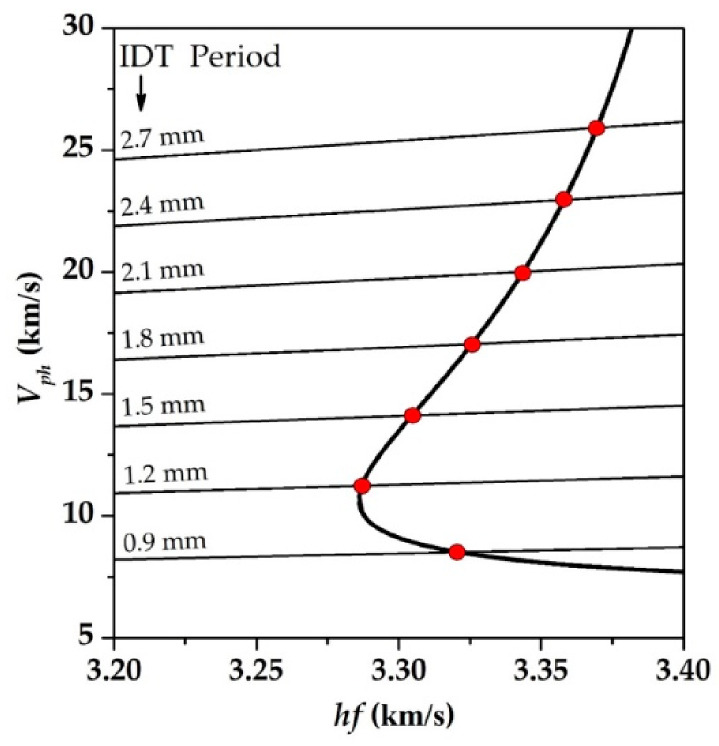
The dependence of the phase velocity of the A_1_ wave in the YX LiNbO_3_ plate (*h* = 350 μm) on the normalized frequency *hf*.

To confirm the results of the theoretical analysis and simulation, an experimental sample containing seven IDTs was fabricated. The IDT parameters were similar to those of the model described above (Section 2.1.2). To select the IDT period, the dependence of the phase velocity of the A_1_ wave in the YX LiNbO_3_ plate on the normalized frequency *hf* was calculated using the method described in Section 2.1.1 (Figure 3).

The straight lines correspond to the selected IDT periods. Their intersection points with the dispersion curve correspond to the expected locations of the resonant peak maxima of the devices under study. The selection of IDT periods from 1.5 mm to 2.7 mm made it possible to evaluate the behavior of the phase velocity in the frequency range where the backward wave exists. At a period of 1.2 mm, the region near the zero-group velocity point (ZGV) was examined, while a period of 0.9 mm corresponded to the excitation of the forward A_1_ wave. The fabrication of transducers with a period of less than 0.9 mm was deemed impractical due to the small electromechanical coupling coefficient of the forward wave at frequencies above 3.325 MHz.

As in the model from Section 2.1.2, each fabricated IDT contained 6 pairs of electrodes and had an aperture of 9 mm [24]. The substrate used was a Y-cut lithium niobate wafer polished on both sides, with a thickness of 350 ± 1 μm (Fomos Materials, Moscow, Russia). The dimensions of each IDT enabled the placement of the necessary number of electrode structures on the wafer within the operating wavelength range.

The electrode structures were fabricated using direct projection photolithography. First, the plate was cleaned with acetone. Then, a layer of photoresist S1813SP15 (Shipley, Sasagami, Japan) with a thickness of 2 μm was applied to its surface. Next, the photoresist was baked for 30 min at a temperature of 94 °C. Photolithography was performed using a Smart-Print system (Microlight 3D, Grenoble, France). The exposed areas of the photoresist were removed using a developer solution P-236A-MF (FRAST-M, Moscow, Russia). A 500 nm thick aluminum layer was deposited onto the plate surface by magnetron sputtering. The deposition process was carried out at a discharge power of 250 W for 8 min, with a chamber pressure of 5.6 × 10^−3^ Torr. The remaining photoresist was removed in an ultrasonic bath using acetone.

To create the liquid cell holder, photopolymer 3D printing technology (Phrozen Sonic Mini 8K, Xiamen City, China) was used. Indium contacts and copper wires with a diameter of 100 μm were used to connect the IDTs to the connectors. BNC connectors fixed at the edges of the holder were used as the connectors.

**Figure 4:**
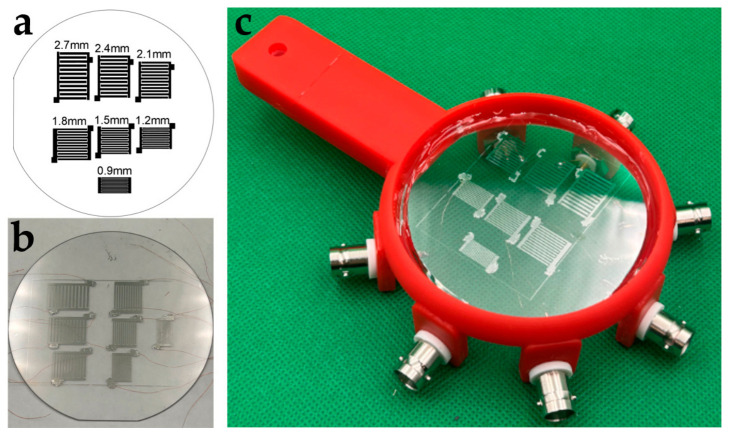
(**a**) Image of the electronic photomask; (**b**) photograph of the fabricated experimental sample with a set of IDTs placed in (**c**) a holder with connectors.

Figure 4 shows an image of the electronic photomask (a), the electrode structure formed on the surface of the piezoelectric plate (b), and the assembled experimental sample fixed in the holder (c).

During the experiment, the thickness of the liquid layer was 5 mm, and it covered the entire area of the plate. It should be noted that the value of the parameter S_11_ at the resonant frequency depended on the thickness of the water layer, and this dependence disappeared only when the thickness of the water layer was more than 3 mm.

#### 2.2.2. Test Liquids

The test liquids used were nonpolar organic solvent chemically pure dichloroethane (DCE) (Sigma-Aldrich, St. Louis, MO, USA), polar liquid ultrapure acetone (Sigma-Aldrich, St. Louis, MO, USA), polar liquid distilled water, and nonpolar liquid 92-octane petrol (“Lukoil”, Moscow, Russia). Table 2 presents the main characteristics of these liquids and air at a temperature of 25 °C [28].

#### 2.2.3. Study of S_11_ Parameter

To investigate the frequency dependence of the *S*_11_ parameter of the fabricated acousto-electronic devices, a vector network analyzer (Tektronix TTR 506A, Beaverton, OR, USA) was used together with a phase-stable cable assembly. Calibration of the vector network analyzer was performed using an OSLT compact calibration kit (4-in-1) DC–9 GHz N-male (SPINNER, Munich, Germany). Then, the IDTs under testing were sequentially connected to it, and the frequency dependence of the *S*_11_ parameter was measured under no-load conditions (in air) and in the presence of the test liquid. All experiments were carried out by means of a thermostat at room temperature (25 °C). The measurement accuracy was ±0.05 dB. It should be noted that the *S*_11_ parameter is the reflection coefficient and characterizes the efficiency of converting electrical energy into acoustic energy and vice versa.

## 3. Results and Discussion

### 3.1. Theoretical Results

**Figure 5:**
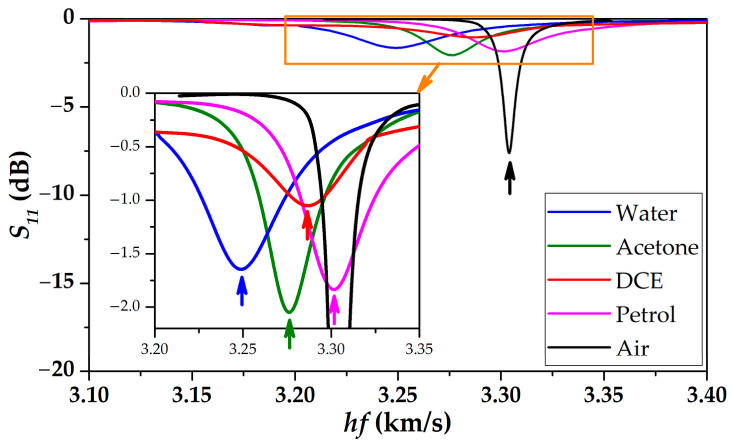
Frequency dependences of the S_11_ parameter for the IDT with a period of 1.5 mm under no-load conditions and in the presence of the test liquids obtained using FEM.

**Figure 6:**
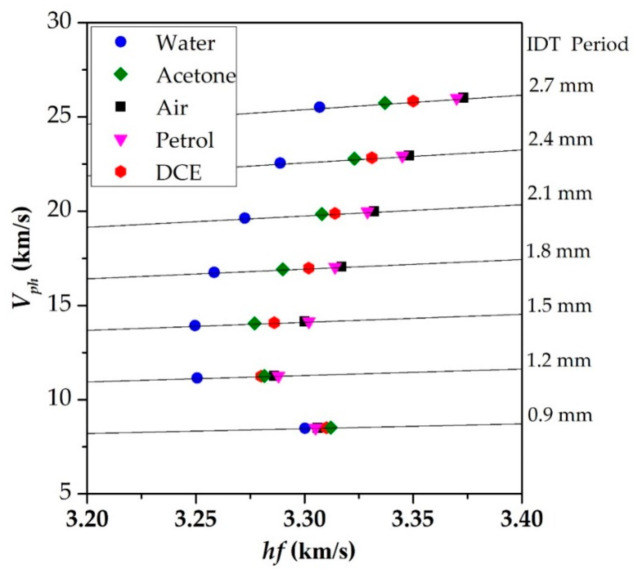
The dependences of the phase velocity of the A_1_ wave in the YX LiNbO_3_ plate on the *hf* obtained using data from FEM analysis.

The dependences of the S_11_ parameter on frequency for all seven IDTs in contact with both air and the test liquids were calculated using the finite element method. As an example, Figure 5 shows the dependence for the IDT with a period of 1.5 mm. Figure 6 shows the dependence of the phase velocity of the A_1_ wave on the *hf* parameter for various values of the IDT period. This dependence was obtained using the expression *V*_ph_ = *λf*, where *λ* and *f* correspond to the wavelength (IDT period) and the frequency of the maximum value of the *S*_11_ parameter, respectively.

Analysis of Figure 5 shows that with an increase in the dielectric permittivity of the liquid, the maxima of the *S*_11_ parameter on the frequency dependence shift toward lower frequencies. This effect is observed for all IDT periods in the range of 1.2–2.7 mm, corresponding to the backward A_1_ wave. Furthermore, a decrease in the absolute value of the *S*_11_ maximum and in the quality factor of the resonant curve is observed.

As can be seen from Table 2, all of the liquids used are characterized by low values of viscosity and electrical conductivity. Their main difference lies in the differences in permittivity and density. It should also be noted that the liquid is located on the back side of the plate in the region of the IDT, and there is a possibility that the liquid permittivity influences the characteristics of the IDT itself.

**Figure 7:**
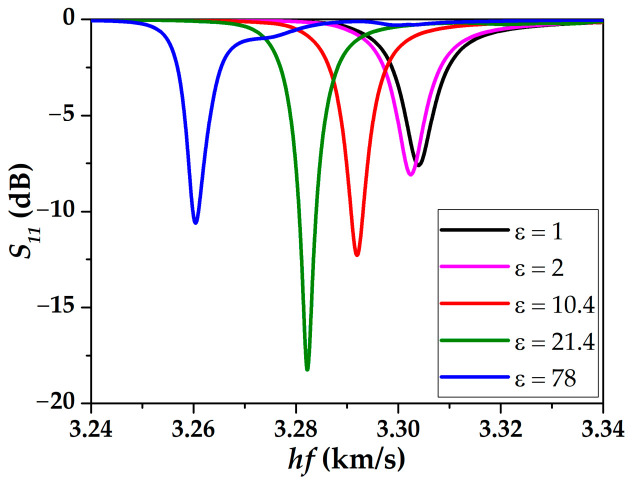
Frequency dependences of the S_11_ parameter for the model structure with different values of the dielectric permittivity of the contacting medium for the IDT with a period of 1.5 mm.

In order to evaluate the influence of only the dielectric permittivity of the liquid on the *S*_11_ parameter, the dependences *S*_11_(*f*) were calculated using FEM for a model structure. This structure consisted of an IDT with a period of 1.5 mm, and the medium used as a load had the mechanical parameters of air but with different values of permittivity. The obtained results are shown in Figure 7.

Comparison of Figure 5 and Figure 7 shows that the shift in the resonant frequency toward lower frequencies is indeed determined by the permittivity of the contacting liquid. This is associated with a partial “short-circuiting” of the piezoelectric surface. The higher the dielectric permittivity of the liquid, the more the piezoelectric effect is “switched off”, and the more the phase velocity decreases.

At the same time, the decrease in the resonance quality factor is associated with the acoustic impedance of the liquid. From Figure 5, it can be seen that the higher the acoustic impedance of the liquid, the lower the quality factor of the resonant curve, and hence, the lower the efficiency of converting electrical energy into acoustic energy and vice versa.

In order to determine how much the permittivity of the liquid affects the characteristics of the IDT itself, rather than the properties of the acoustic wave itself, the phase velocity of the wave was calculated for a model structure using the transfer matrix method. The phase velocity of the A_1_ wave, taking into account its excitation by the IDT, was determined from Figure 7 using the formula *V*_ph_ = *λf*. Table 3 presents the comparison results.

It can be seen that with an increase in the dielectric permittivity of the medium, the relative difference between the phase velocities of the A_1_ wave calculated with and without excitation taken into account increases, but by no more than 1%. This indicates that the dielectric permittivity of the medium located above the IDT affects its characteristics; however, this effect is negligible.

### 3.2. Experimental Results

**Figure 8:**
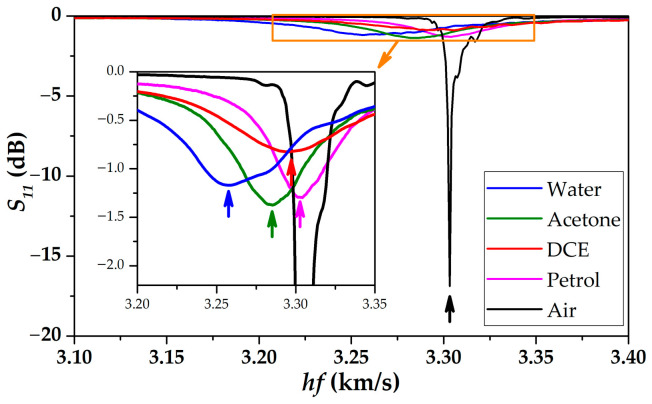
Frequency dependences of the S_11_ parameter for the IDT with a period of 1.5 mm under no-load conditions and in the presence of the test liquids obtained experimentally.

**Figure 9:**
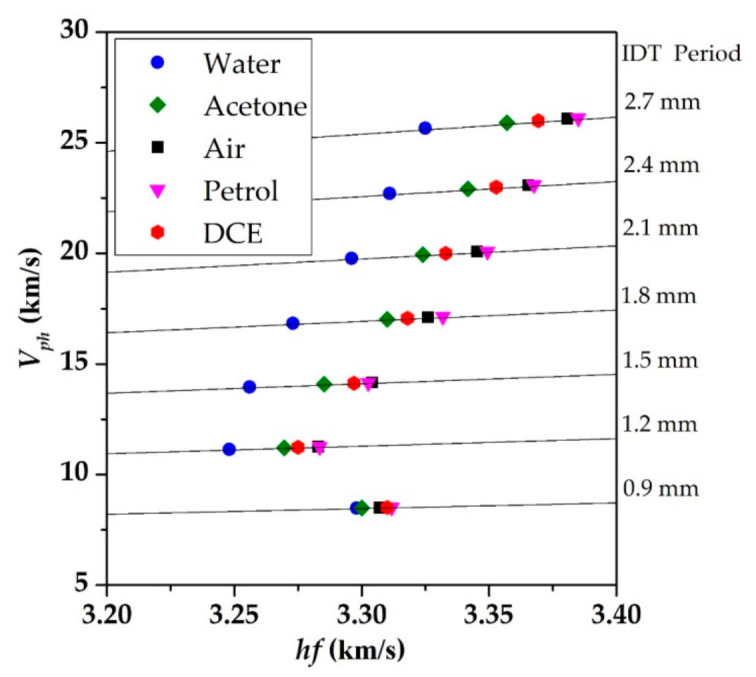
The dependences of the phase velocity of the A_1_ wave in the YX LiNbO_3_ plate on the *hf* obtained using experimental data.

The dependences of the *S*_11_ parameter on frequency were measured for all fabricated IDTs with periods in the range of 0.9–2.7 mm. First, the dependences were measured in the presence of air, and then in the presence of water, acetone, DCE, and petrol. Figure 8 shows, as an example, the measured frequency dependences of the *S*_11_ parameter for the IDT with a period of 1.5 mm under no-load conditions and in the presence of the test liquids. Figure 9 shows the dependence of the phase velocity of the A_1_ wave on the *hf* parameter obtained from the experimental results using the expression *V*_ph_ = *λf*. Comparison of Figure 5 and Figure 8, as well as Figure 6 and Figure 9, shows good qualitative and quantitative agreement between the theoretical and experimental results. The calculated and measured resonant frequencies agree particularly well. It should be noted that there is a difference between the theoretically obtained and experimentally measured values of the *S*_11_ parameter upon contact with air. This can be explained by the presence of acoustic modes reflected from the edges of the plate, which, when added in phase, can increase the value of the *S*_11_ parameter. The appearance of a load on the opposite side of the plate suppresses these reflected signals, which, in the experiment, leads to a significant decrease in the amplitude of the S_11_ parameter.

Analysis of Figure 8 and Figure 9 confirms the conclusions of the theory. It can be seen that the predominant influence on the position of the maxima is exerted by the value of the dielectric permittivity, i.e., the liquid acts as a dielectric load on the electric field accompanying the acoustic wave. The higher its dielectric permittivity, the more electrostatic energy is stored in the liquid, which affects the effective velocity and the resonance conditions. However, the magnitude of the resonant peak appears to be determined by acoustic losses. As previously noted for this type of device, the greater the depth of the peak in the frequency dependence of the *S*_11_ parameter, the more efficiently the wave is excited. Since the *S*_11_ parameter characterizes the reflection coefficient, the deeper the peak, the more energy is radiated by the acousto-electronic device at a given frequency. The liquid loading, overall, leads to a significant reduction in the magnitude of the resonant peak due to high radiation losses associated with the wave type and the magnitude of the mechanical displacement component perpendicular to the surface of the piezoelectric plate. In this regard, the intensity of radiation is determined primarily by the acoustic impedance of the liquid at the interface. The heights of the peaks correlate with the values of the acoustic impedances presented in Table 1. Water and DCE have the greatest influence, with impedance values of 1.49 × 10^6^ kg/m^2^·s and 1.44 × 10^6^ kg/m^2^·s, respectively, whereas acetone and petrol, with values of 0.92 × 10^6^ kg/m^2^·s and 0.98 × 10^6^ kg/m^2^·s, have a weaker effect on the excitation efficiency.

## 4. Conclusions

Thus, in this work, a theoretical, numerical, and experimental study was carried out on the influence of liquids with various acoustic impedances and dielectric permittivities on the characteristics of the backward antisymmetric Lamb wave A_1_ in a YX LiNbO_3_ plate. It was established that the effect of the liquid on the characteristics of the backward A_1_ wave has a dual nature. First, an increase in the dielectric permittivity of the liquid leads to a decrease in the resonant frequency and, accordingly, in the phase velocity of the wave. This effect arises due to an increase in the fraction of electrical energy stored in the liquid at the expense of a decrease in the mechanical energy of the acoustic wave. This leads to a reduction in the effective stiffness of the plate and, as a consequence, a decrease in the phase velocity. Second, an increase in the acoustic impedance of the liquid leads to a decrease in both the amplitude of the S_11_ parameter and the quality factor of the resonant curve. This is explained by the fact that the antisymmetric A_1_ wave possesses a substantial normal component of mechanical displacement at the plate surface, which results in efficient radiation of acoustic energy into the liquid. The higher the acoustic impedance of the liquid, the more energy is transferred from the plate into the liquid, which manifests itself in a decrease in the quality factor and an increase in insertion loss.

One of the main results of this work is the quantitative separation of the influence of the dielectric permittivity and the acoustic impedance of the liquid. Using a specially developed FEM model, in which the mechanical parameters of the liquid were set equal to those of air while varying only the dielectric permittivity, it was shown that the shift in the resonant frequency is almost entirely determined by the dielectric properties of the medium. Moreover, the change in phase velocity calculated by taking into account wave excitation by means of the IDT (FEM) and without it (transfer matrix method) does not exceed 1%, even for water with *ε* = 78.5. This proves that the dielectric permittivity of the liquid affects the device characteristics primarily through modification of the electric field of the wave itself, rather than through changes in the parameters of the IDT as an electrode structure. This allows us to suggest that the resonant frequency shift can be interpreted directly as a measure of the dielectric permittivity of the non-conductive and inviscid liquid without the need to introduce additional corrections for the design features of the transducer. When developing a practical sensor, a one-time calibration using a liquid with a known dielectric permittivity can be performed for each specific IDT. This automatically accounts for small corrections related to the device design. Then, for the remaining liquids, one can use the direct dependence of the frequency shift on the dielectric permittivity without additional corrections for excitation, as confirmed by our experiment.

It should also be noted that for liquids with extremely high density and simultaneously high permittivity (e.g., certain ionic liquids), a more complex calibration may be required.

The comparison of theoretical and experimental results showed good qualitative and quantitative agreement. The calculated and measured resonant frequencies agree to within 1–2%. Slight discrepancies in the absolute values of the S_11_ parameter can be explained by the presence of spurious reflected acoustic modes from the edges of the plate in the experiment, which are absent in the FEM model due to the perfectly matched layers. It is important to note that when the liquid is applied, the reflected signals are suppressed, and the experimental resonant curves become closer to the calculated ones, which confirms the validity of the simulation.

The obtained results open up prospects for using the backward A_1_ wave in YX LiNbO_3_ to create a two-parameter acousto-electronic sensor for liquid properties, capable of simultaneously and independently measuring its dielectric permittivity and density (or acoustic impedance). Such a device would be in demand for determining the grade of petrol or diesel fuel by the pair of values (*ε* and *ρ*), detecting adulteration or dilution, analyzing binary mixtures, and monitoring the condition of industrial liquids.

For the practical implementation of a two-parameter sensor based on the backward A_1_ wave in a YX LiNbO_3_ plate, it is necessary to ensure independent determination of the dielectric permittivity and acoustic impedance (density) of the liquid. As shown in the work, the resonance frequency shift is determined primarily by the dielectric permittivity of the medium and is practically independent of the acoustic load, while the change in the quality factor (or the depth of the resonance dip of the S_11_ parameter) is mainly related to the acoustic impedance of the liquid. Due to the orthogonality of these responses, unambiguous separation of the parameters is possible. The following calibration and measurement procedure is proposed.

At the first stage, using two reference liquids with known values of dielectric permittivity and acoustic impedance (e.g., water and acetone), two calibration curves are constructed for a specific interdigital transducer with a fixed period: the dependence of the resonance frequency on dielectric permittivity (with the averaged influence of impedance) and the dependence of the inverse quality factor (or peak amplitude) on acoustic impedance. Since the influence of impedance on the frequency shift does not exceed the measurement error, the first curve can be approximated by a monotonic function relating frequency and dielectric permittivity. Similarly, for the quality factor, the influence of dielectric permittivity is negligible in the range of ε from 2 to 80.

At the second stage, the resonance frequency and quality factor are measured for the test liquid. The dielectric permittivity is determined from the frequency using the first calibration curve. Then, by substituting the obtained permittivity value into the second calibration curve (or using an independent dependence of the quality factor on impedance constructed at a fixed ε), the acoustic impedance of the liquid is found. Such a two-stage procedure completely eliminates the ambiguity in parameter determination and does not require complex mathematical processing. If higher accuracy is needed, an iterative scheme accounting for small cross-effects can be applied. However, for most practical applications (fuel identification, binary mixture analysis, and industrial liquid monitoring), the described approach is sufficient.

The obtained results show that the proposed method allows simultaneous and independent measurement of both dielectric permittivity and density (speed of sound) of inviscid non-conductive liquids with an error not exceeding 2–3% for each parameter.

## Figures and Tables

**Table 1:** The material constants of LiNbO_3_ crystal [24].

Elastic Moduli, *C*^E^_ij_ (10^10^ N/m^2^)						
*C* ^E^_11_	*C* ^E^_12_	*C* ^E^_13_	*C* ^E^_14_	*C* ^E^_33_	*C* ^E^_44_	*C* ^E^_66_
20.3	5.73	7.52	0.85	24.24	5.95	7.28
Piezoconstants, *e*_ij_ (C/m^2^)				Dielectric Permittivity, *ε*^S^_ij_/*ε*_0_		Density, kg/m^3^
*e*_15_	*e*_22_	*e*_31_	*e*_33_	*ε* ^S^_11_	*ε* ^S^_33_	*ρ*
3.83	2.37	0.23	1.3	44.3	27.9	4650

**Table 2:** Basic physical properties of air and the studied liquids [28].

Liquid	Density (kg/m^3^)	Relative Permittivity	Speed of Sound (m/s)	Acoustic Impedance (×10^6^ kg/m^2^·s)	Longitudinal Elastic Constant (GPa)	Dynamic Viscosity (mPa·s)	Conductivity (µS/cm)
Water	997	78.5	1498	1.49	2.25	0.89	0.5–5.5
Acetone	784	20.7	1174	0.92	0.92	0.31	0.02–0.06
DCE	1250	10.36	1150	1.44	1.51	0.9	0.001–0.1
Petrol	760	2.0	1290	0.98	1.04	0.5	<0.001
Air	1.184	1.006	346	0.00041	0.000142	0.0184	0.00004

**Table 3:** Comparison of the phase velocity of the A_1_ wave in YX LiNbO_3_ plate calculated with (FEM) and without (TMM) excitation taken into account.

ε/ε_0_	Transfer Matrix Method		FEM, IDT Period 1.5 mm		$\frac{\left(V_{ph}^{FEM}-V_{ph}^{TMM}\right)}{V_{ph}^{TMM}}$,%
	hf (km/s)	$V_{ph}^{TMM}$ (km/s)	hf (km/s)	$V_{ph}^{FEM}$ (km/s)	
1	3.304	14.122	3.304	14.159	0.26
2	3.303	14.116	3.302	14.152	0.26
10.36	3.289	14.052	3.292	14.108	0.40
20.7	3.277	14.001	3.282	14.066	0.46
78.5	3.243	13.859	3.260	13.971	0.80

## Data Availability

The original contributions presented in this study are included in the article. Further inquiries can be directed to the corresponding author.
